# 2-Chloro-1-(4-hy­droxy­phen­yl)ethanone

**DOI:** 10.1107/S1600536812030838

**Published:** 2012-07-10

**Authors:** Hoong-Kun Fun, Ching Kheng Quah, Divya N. Shetty, B. Narayana, B. K. Sarojini

**Affiliations:** a X-ray Crystallography Unit, School of Physics, Universiti Sains Malaysia, 11800 USM, Penang, Malaysia; b Department of Studies in Chemistry, Mangalore University, Mangalagangotri 574 199, India; c Department of Chemistry, P. A. College of Engineering, Nadupadavu, Mangalore 574 153, India

## Abstract

The asymmetric unit of the title compound, C_8_H_7_ClO_2_, consists of two independent mol­ecules, with comparable geometries. Both mol­ecules are approximately planar (r.m.s. deviations = 0.040 and 0.064 Å for the 11 non-H atoms). In the crystal, mol­ecules are linked *via* inter­molecular O—H⋯O and C—H⋯O hydrogen bonds into chains two mol­ecules thick along (-101).

## Related literature

For general background to and related structures of the title compound, see: Erian *et al.* (2003 ▶); Qing & Zhang (2009 ▶); Fun *et al.* (2012 ▶). For standard bond-length data, see: Allen *et al.* (1987 ▶). For the stability of the temperature controller used for the data collection, see: Cosier & Glazer (1986 ▶).
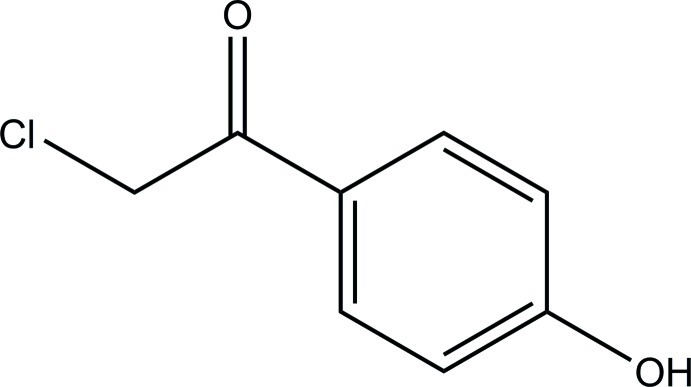


## Experimental

### 

#### Crystal data

C_8_H_7_ClO_2_
*M* *_r_* = 170.59
Monoclinic, 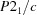

*a* = 7.4931 (5) Å
*b* = 14.7345 (10) Å
*c* = 13.5681 (10) Å
β = 95.560 (1)°
*V* = 1490.97 (18) Å^3^
*Z* = 8
Mo *K*α radiation
μ = 0.45 mm^−1^
*T* = 100 K
0.51 × 0.23 × 0.18 mm

#### Data collection

Bruker SMART APEXII DUO CCD area-detector diffractometer
Absorption correction: multi-scan (*SADABS*; Bruker, 2009 ▶) *T*_min_ = 0.803, *T*_max_ = 0.925
16799 measured reflections
4352 independent reflections
4037 reflections with *I* > 2σ(*I*)
*R*_int_ = 0.019

#### Refinement

*R*[*F* ^2^ > 2σ(*F* ^2^)] = 0.026
*wR*(*F* ^2^) = 0.073
*S* = 1.03
4352 reflections
207 parameters
H atoms treated by a mixture of independent and constrained refinement
Δρ_max_ = 0.46 e Å^−3^
Δρ_min_ = −0.23 e Å^−3^

### 

Data collection: *APEX2* (Bruker, 2009 ▶); cell refinement: *SAINT* (Bruker, 2009 ▶); data reduction: *SAINT*; program(s) used to solve structure: *SHELXTL* (Sheldrick, 2008 ▶); program(s) used to refine structure: *SHELXTL*; molecular graphics: *SHELXTL*; software used to prepare material for publication: *SHELXTL* and *PLATON* (Spek, 2009 ▶).

## Supplementary Material

Crystal structure: contains datablock(s) global, I. DOI: 10.1107/S1600536812030838/sj5254sup1.cif

Structure factors: contains datablock(s) I. DOI: 10.1107/S1600536812030838/sj5254Isup2.hkl

Supplementary material file. DOI: 10.1107/S1600536812030838/sj5254Isup3.cml

Additional supplementary materials:  crystallographic information; 3D view; checkCIF report

## Figures and Tables

**Table 1:** Hydrogen-bond geometry (Å, °)

*D*—H⋯*A*	*D*—H	H⋯*A*	*D*⋯*A*	*D*—H⋯*A*
O1*B*—H2*O*1⋯O2*A* ^i^	0.812 (16)	1.973 (16)	2.7742 (11)	168.7 (16)
O1*A*—H1*O*1⋯O2*B* ^ii^	0.840 (16)	1.891 (16)	2.7229 (10)	170.4 (16)
C4*A*—H4*AA*⋯O2*B* ^ii^	0.95	2.47	3.1780 (12)	131
C8*A*—H8*AA*⋯O1*A* ^iii^	0.99	2.58	3.5228 (12)	160
C2*B*—H2*BA*⋯O2*A* ^i^	0.95	2.44	3.1603 (12)	133
C4*B*—H4*BA*⋯O1*A* ^iv^	0.95	2.58	3.5126 (12)	166

Symmetry codes: (i) 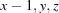; (ii) 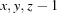; (iii) 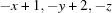; (iv) 
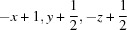
.

**Fig. 1.**
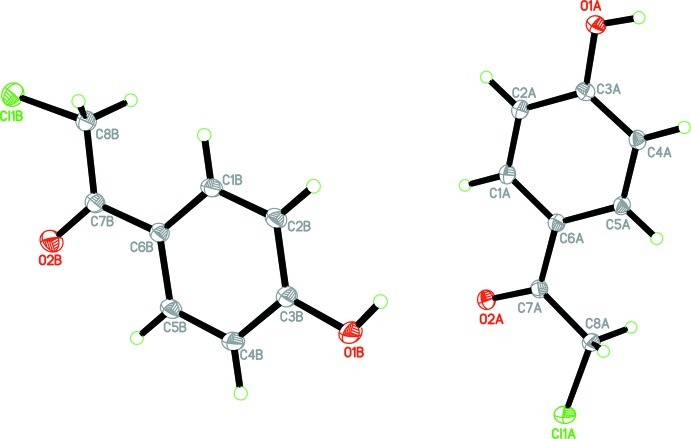
Fig. 1. The asymmetric unit of the title compound showing 50% probability displacement ellipsoids for non-H atoms.

**Fig. 2.**
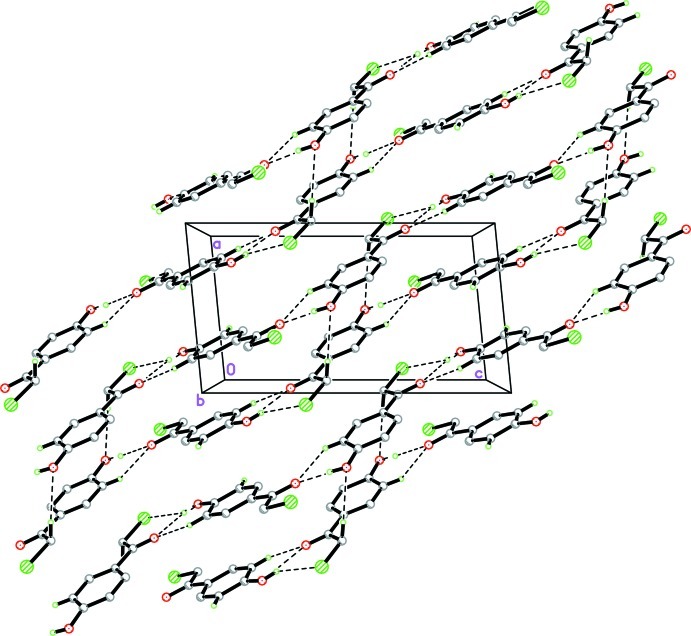
The crystal structure of the title compound, viewed along the b axis. H atoms not involved in hydrogen bonds (dashed lines) have been omitted for clarity.

## supplementary crystallographic information

### Comment

In view of the importance of *α*-haloketones in heterocyclic synthesis and their reactivity towards oxygen nucleophiles (Erian *et al.*, 2003), the crystal structure of title compond (I) is reported. The crystal structure of the bromo analogue of the title compound has also been reported (Qing & Zhang, 2009).

The asymmetric unit (Fig. 1) of the title compound consists of two independent molecules (*A* and *B*), with comparable geometries. Both molecules (*A* and *B*) are approximately planar (r.m.s. deviation = 0.040 and 0.064 Å, respectively, for the eleven non-H atoms). Bond lengths (Allen *et al.*, 1987) and angles are within normal ranges and are comparable with a related structure (Fun *et al.*, 2012).

In the crystal structure, Fig. 2, molecules are linked *via* intermolecular O1B–H1O2···O2A, O1A–H1O1···O2B, C4A–H4AA···O2B, C8A–H8AA···O1A, C2B–H2BA···O2A and C4B–H4BA···O1A hydrogen bonds (Table 1) into two-molecular-thick chains along the [-101].

### Experimental

The title compound, 2-chloro-1-(4-hydroxyphenyl)ethanone, was purchased from Sigma-Aldrich and recrystallized from methanol by the slow evaporation method (*m.p.* 422 K).

### Refinement

O-bound hydrogen atoms were located in a difference Fourier map and refined freely with O–H = 0.814 (17) - 0.840 (17) Å. The remaining H atoms were positioned geometrically and refined using a riding model with C–H = 0.95 or 0.99 Å and *U*_iso_(H) = 1.2 *U*_eq_(C).

### Crystal data

**Table:**

C_8_H_7_ClO_2_	*F*(000) = 704
*M**_r_* = 170.59	*D*_x_ = 1.520 Mg m^−^^3^
Monoclinic, *P*2_1_/*c*	Mo *K*α radiation, λ = 0.71073 Å
Hall symbol: -P 2ybc	Cell parameters from 9970 reflections
*a* = 7.4931 (5) Å	θ = 2.7–30.1°
*b* = 14.7345 (10) Å	µ = 0.45 mm^−^^1^
*c* = 13.5681 (10) Å	*T* = 100 K
β = 95.560 (1)°	Block, colourless
*V* = 1490.97 (18) Å^3^	0.51 × 0.23 × 0.18 mm
*Z* = 8	

### Data collection

**Table:**

Bruker SMART APEXII DUO CCD area-detector diffractometer	4352 independent reflections
Radiation source: fine-focus sealed tube	4037 reflections with *I* > 2σ(*I*)
Graphite monochromator	*R*_int_ = 0.019
φ and ω scans	θ_max_ = 30.1°, θ_min_ = 2.7°
Absorption correction: multi-scan (*SADABS*; Bruker, 2009)	*h* = −9→10
*T*_min_ = 0.803, *T*_max_ = 0.925	*k* = −20→20
16799 measured reflections	*l* = −19→17

### Refinement

**Table:**

Refinement on *F*^2^	Primary atom site location: structure-invariant direct methods
Least-squares matrix: full	Secondary atom site location: difference Fourier map
*R*[*F*^2^ > 2σ(*F*^2^)] = 0.026	Hydrogen site location: inferred from neighbouring sites
*wR*(*F*^2^) = 0.073	H atoms treated by a mixture of independent and constrained refinement
*S* = 1.03	*w* = 1/[σ^2^(*F*_o_^2^) + (0.037*P*)^2^ + 0.5857*P*] where *P* = (*F*_o_^2^ + 2*F*_c_^2^)/3
4352 reflections	(Δ/σ)_max_ = 0.003
207 parameters	Δρ_max_ = 0.46 e Å^−^^3^
0 restraints	Δρ_min_ = −0.23 e Å^−^^3^

### Special details

**Table:**

Experimental. The crystal was placed in the cold stream of an Oxford Cryosystems Cobra open-flow nitrogen cryostat (Cosier & Glazer, 1986) operating at 100.0 (1) K.
Geometry. All esds (except the esd in the dihedral angle between two l.s. planes) are estimated using the full covariance matrix. The cell esds are taken into account individually in the estimation of esds in distances, angles and torsion angles; correlations between esds in cell parameters are only used when they are defined by crystal symmetry. An approximate (isotropic) treatment of cell esds is used for estimating esds involving l.s. planes.
Refinement. Refinement of F^2^ against ALL reflections. The weighted R-factor wR and goodness of fit S are based on F^2^, conventional R-factors R are based on F, with F set to zero for negative F^2^. The threshold expression of F^2^ > 2sigma(F^2^) is used only for calculating R-factors(gt) etc. and is not relevant to the choice of reflections for refinement. R-factors based on F^2^ are statistically about twice as large as those based on F, and R- factors based on ALL data will be even larger.

### Fractional atomic coordinates and isotropic or equivalent isotropic displacement parameters (Å^2^)

**Table:**

	*x*	*y*	*z*	*U*_iso_*/*U*_eq_	
Cl1A	1.09654 (3)	1.232822 (15)	0.175736 (18)	0.02002 (7)	
O1A	0.51043 (9)	0.80027 (5)	−0.05945 (5)	0.01637 (13)	
O2A	1.01029 (10)	1.04907 (5)	0.22919 (5)	0.01973 (14)	
C1A	0.79616 (12)	0.91436 (6)	0.13312 (7)	0.01573 (17)	
H1AA	0.8461	0.9029	0.1990	0.019*	
C2A	0.69221 (12)	0.84846 (6)	0.08224 (7)	0.01577 (17)	
H2AA	0.6716	0.7920	0.1130	0.019*	
C3A	0.61778 (12)	0.86551 (6)	−0.01468 (7)	0.01370 (16)	
C4A	0.65279 (12)	0.94739 (6)	−0.06163 (7)	0.01386 (16)	
H4AA	0.6060	0.9580	−0.1282	0.017*	
C5A	0.75661 (12)	1.01286 (6)	−0.00971 (7)	0.01353 (16)	
H5AA	0.7794	1.0687	−0.0411	0.016*	
C6A	0.82845 (11)	0.99793 (6)	0.08835 (7)	0.01357 (16)	
C7A	0.93813 (12)	1.06615 (6)	0.14663 (7)	0.01399 (16)	
C8A	0.95428 (12)	1.15941 (6)	0.10015 (7)	0.01518 (16)	
H8AA	0.8336	1.1870	0.0887	0.018*	
H8AB	1.0022	1.1526	0.0351	0.018*	
Cl1B	0.32249 (4)	0.674926 (16)	0.744284 (18)	0.02232 (7)	
O1B	0.21395 (11)	1.12541 (5)	0.38976 (6)	0.02120 (15)	
O2B	0.39854 (10)	0.86748 (5)	0.75803 (5)	0.02074 (15)	
C1B	0.20436 (12)	0.90119 (6)	0.50470 (7)	0.01514 (16)	
H1BA	0.1632	0.8413	0.4897	0.018*	
C2B	0.17605 (12)	0.96844 (6)	0.43372 (7)	0.01547 (17)	
H2BA	0.1156	0.9550	0.3706	0.019*	
C3B	0.23734 (12)	1.05637 (6)	0.45581 (7)	0.01522 (17)	
C4B	0.32607 (13)	1.07671 (6)	0.54895 (7)	0.01661 (17)	
H4BA	0.3674	1.1366	0.5636	0.020*	
C5B	0.35289 (12)	1.00894 (6)	0.61929 (7)	0.01579 (17)	
H5BA	0.4126	1.0227	0.6825	0.019*	
C6B	0.29280 (12)	0.91996 (6)	0.59837 (7)	0.01388 (16)	
C7B	0.32576 (12)	0.85017 (6)	0.67533 (7)	0.01461 (16)	
C8B	0.26334 (13)	0.75424 (6)	0.64795 (7)	0.01729 (17)	
H8BA	0.3173	0.7350	0.5876	0.021*	
H8BB	0.1314	0.7543	0.6327	0.021*	
H2O1	0.162 (2)	1.1077 (11)	0.3380 (12)	0.034 (4)*	
H1O1	0.480 (2)	0.8153 (11)	−0.1184 (12)	0.032 (4)*	

### Atomic displacement parameters (Å^2^)

**Table:**

	*U*^11^	*U*^22^	*U*^33^	*U*^12^	*U*^13^	*U*^23^
Cl1A	0.02376 (12)	0.01545 (11)	0.02045 (12)	−0.00383 (8)	0.00009 (8)	−0.00221 (8)
O1A	0.0183 (3)	0.0151 (3)	0.0150 (3)	−0.0022 (2)	−0.0020 (2)	−0.0001 (2)
O2A	0.0253 (3)	0.0182 (3)	0.0145 (3)	−0.0026 (3)	−0.0043 (3)	0.0019 (3)
C1A	0.0188 (4)	0.0149 (4)	0.0130 (4)	0.0001 (3)	−0.0006 (3)	0.0023 (3)
C2A	0.0190 (4)	0.0141 (4)	0.0140 (4)	−0.0002 (3)	0.0003 (3)	0.0025 (3)
C3A	0.0130 (3)	0.0136 (4)	0.0143 (4)	0.0010 (3)	0.0008 (3)	−0.0012 (3)
C4A	0.0146 (4)	0.0147 (4)	0.0120 (4)	0.0017 (3)	−0.0001 (3)	0.0009 (3)
C5A	0.0148 (4)	0.0126 (4)	0.0131 (4)	0.0011 (3)	0.0010 (3)	0.0013 (3)
C6A	0.0150 (4)	0.0129 (4)	0.0127 (4)	0.0007 (3)	0.0008 (3)	0.0005 (3)
C7A	0.0152 (4)	0.0139 (4)	0.0129 (4)	0.0010 (3)	0.0014 (3)	0.0002 (3)
C8A	0.0167 (4)	0.0136 (4)	0.0149 (4)	−0.0005 (3)	−0.0005 (3)	0.0007 (3)
Cl1B	0.03273 (13)	0.01561 (11)	0.01846 (12)	0.00168 (8)	0.00162 (9)	0.00428 (8)
O1B	0.0316 (4)	0.0143 (3)	0.0161 (3)	−0.0032 (3)	−0.0060 (3)	0.0030 (3)
O2B	0.0284 (4)	0.0187 (3)	0.0139 (3)	−0.0009 (3)	−0.0047 (3)	0.0002 (3)
C1B	0.0186 (4)	0.0123 (4)	0.0141 (4)	−0.0009 (3)	−0.0008 (3)	−0.0017 (3)
C2B	0.0189 (4)	0.0141 (4)	0.0127 (4)	0.0001 (3)	−0.0021 (3)	−0.0012 (3)
C3B	0.0180 (4)	0.0131 (4)	0.0142 (4)	0.0002 (3)	0.0001 (3)	0.0006 (3)
C4B	0.0203 (4)	0.0131 (4)	0.0159 (4)	−0.0025 (3)	−0.0009 (3)	−0.0018 (3)
C5B	0.0185 (4)	0.0150 (4)	0.0132 (4)	−0.0014 (3)	−0.0017 (3)	−0.0020 (3)
C6B	0.0160 (4)	0.0129 (4)	0.0125 (4)	0.0001 (3)	0.0002 (3)	−0.0004 (3)
C7B	0.0164 (4)	0.0140 (4)	0.0133 (4)	0.0003 (3)	0.0010 (3)	−0.0007 (3)
C8B	0.0240 (4)	0.0135 (4)	0.0139 (4)	−0.0004 (3)	−0.0003 (3)	0.0013 (3)

### Geometric parameters (Å, º)

**Table:**

Cl1A—C8A	1.7738 (10)	Cl1B—C8B	1.7772 (10)
O1A—C3A	1.3585 (11)	O1B—C3B	1.3559 (11)
O1A—H1O1	0.840 (17)	O1B—H2O1	0.814 (17)
O2A—C7A	1.2219 (11)	O2B—C7B	1.2260 (12)
C1A—C2A	1.3863 (13)	C1B—C2B	1.3838 (13)
C1A—C6A	1.4043 (12)	C1B—C6B	1.4027 (13)
C1A—H1AA	0.9500	C1B—H1BA	0.9500
C2A—C3A	1.4005 (13)	C2B—C3B	1.3973 (13)
C2A—H2AA	0.9500	C2B—H2BA	0.9500
C3A—C4A	1.4009 (12)	C3B—C4B	1.4021 (13)
C4A—C5A	1.3879 (12)	C4B—C5B	1.3824 (13)
C4A—H4AA	0.9500	C4B—H4BA	0.9500
C5A—C6A	1.4034 (12)	C5B—C6B	1.4062 (12)
C5A—H5AA	0.9500	C5B—H5BA	0.9500
C6A—C7A	1.4783 (12)	C6B—C7B	1.4693 (12)
C7A—C8A	1.5217 (13)	C7B—C8B	1.5235 (13)
C8A—H8AA	0.9900	C8B—H8BA	0.9900
C8A—H8AB	0.9900	C8B—H8BB	0.9900
			
C3A—O1A—H1O1	109.5 (11)	C3B—O1B—H2O1	110.5 (12)
C2A—C1A—C6A	120.72 (9)	C2B—C1B—C6B	121.09 (8)
C2A—C1A—H1AA	119.6	C2B—C1B—H1BA	119.5
C6A—C1A—H1AA	119.6	C6B—C1B—H1BA	119.5
C1A—C2A—C3A	119.69 (8)	C1B—C2B—C3B	119.32 (8)
C1A—C2A—H2AA	120.2	C1B—C2B—H2BA	120.3
C3A—C2A—H2AA	120.2	C3B—C2B—H2BA	120.3
O1A—C3A—C2A	117.23 (8)	O1B—C3B—C2B	122.35 (8)
O1A—C3A—C4A	122.34 (8)	O1B—C3B—C4B	117.07 (8)
C2A—C3A—C4A	120.43 (8)	C2B—C3B—C4B	120.58 (8)
C5A—C4A—C3A	119.23 (8)	C5B—C4B—C3B	119.50 (8)
C5A—C4A—H4AA	120.4	C5B—C4B—H4BA	120.3
C3A—C4A—H4AA	120.4	C3B—C4B—H4BA	120.3
C4A—C5A—C6A	121.12 (8)	C4B—C5B—C6B	120.79 (9)
C4A—C5A—H5AA	119.4	C4B—C5B—H5BA	119.6
C6A—C5A—H5AA	119.4	C6B—C5B—H5BA	119.6
C5A—C6A—C1A	118.77 (8)	C1B—C6B—C5B	118.72 (8)
C5A—C6A—C7A	122.87 (8)	C1B—C6B—C7B	122.54 (8)
C1A—C6A—C7A	118.36 (8)	C5B—C6B—C7B	118.73 (8)
O2A—C7A—C6A	121.60 (8)	O2B—C7B—C6B	122.28 (8)
O2A—C7A—C8A	121.34 (8)	O2B—C7B—C8B	121.00 (8)
C6A—C7A—C8A	117.05 (8)	C6B—C7B—C8B	116.72 (8)
C7A—C8A—Cl1A	112.24 (7)	C7B—C8B—Cl1B	112.46 (7)
C7A—C8A—H8AA	109.2	C7B—C8B—H8BA	109.1
Cl1A—C8A—H8AA	109.2	Cl1B—C8B—H8BA	109.1
C7A—C8A—H8AB	109.2	C7B—C8B—H8BB	109.1
Cl1A—C8A—H8AB	109.2	Cl1B—C8B—H8BB	109.1
H8AA—C8A—H8AB	107.9	H8BA—C8B—H8BB	107.8
			
C6A—C1A—C2A—C3A	−0.33 (14)	C6B—C1B—C2B—C3B	0.25 (14)
C1A—C2A—C3A—O1A	−177.08 (8)	C1B—C2B—C3B—O1B	−179.94 (9)
C1A—C2A—C3A—C4A	2.21 (13)	C1B—C2B—C3B—C4B	−0.25 (14)
O1A—C3A—C4A—C5A	176.85 (8)	O1B—C3B—C4B—C5B	179.73 (9)
C2A—C3A—C4A—C5A	−2.41 (13)	C2B—C3B—C4B—C5B	0.03 (14)
C3A—C4A—C5A—C6A	0.73 (13)	C3B—C4B—C5B—C6B	0.21 (14)
C4A—C5A—C6A—C1A	1.11 (13)	C2B—C1B—C6B—C5B	−0.02 (14)
C4A—C5A—C6A—C7A	−179.37 (8)	C2B—C1B—C6B—C7B	−179.75 (9)
C2A—C1A—C6A—C5A	−1.32 (13)	C4B—C5B—C6B—C1B	−0.21 (14)
C2A—C1A—C6A—C7A	179.14 (8)	C4B—C5B—C6B—C7B	179.53 (9)
C5A—C6A—C7A—O2A	−174.44 (9)	C1B—C6B—C7B—O2B	−177.86 (9)
C1A—C6A—C7A—O2A	5.08 (13)	C5B—C6B—C7B—O2B	2.41 (14)
C5A—C6A—C7A—C8A	6.77 (12)	C1B—C6B—C7B—C8B	1.60 (13)
C1A—C6A—C7A—C8A	−173.71 (8)	C5B—C6B—C7B—C8B	−178.13 (8)
O2A—C7A—C8A—Cl1A	3.57 (11)	O2B—C7B—C8B—Cl1B	−4.04 (12)
C6A—C7A—C8A—Cl1A	−177.63 (6)	C6B—C7B—C8B—Cl1B	176.49 (7)

### Hydrogen-bond geometry (Å, º)

**Table:**

*D*—H···*A*	*D*—H	H···*A*	*D*···*A*	*D*—H···*A*
O1*B*—H2*O*1···O2*A*^i^	0.812 (16)	1.973 (16)	2.7742 (11)	168.7 (16)
O1*A*—H1*O*1···O2*B*^ii^	0.840 (16)	1.891 (16)	2.7229 (10)	170.4 (16)
C4*A*—H4*AA*···O2*B*^ii^	0.95	2.47	3.1780 (12)	131
C8*A*—H8*AA*···O1*A*^iii^	0.99	2.58	3.5228 (12)	160
C2*B*—H2*BA*···O2*A*^i^	0.95	2.44	3.1603 (12)	133
C4*B*—H4*BA*···O1*A*^iv^	0.95	2.58	3.5126 (12)	166

Symmetry codes: (i) *x*−1, *y*, *z*; (ii) *x*, *y*, *z*−1; (iii) −*x*+1, −*y*+2, −*z*; (iv) −*x*+1, *y*+1/2, −*z*+1/2.
